# Fungal fuel cells: an environmentally friendly approach to addressing heavy metal pollution and electricity production

**DOI:** 10.3389/fmicb.2026.1825368

**Published:** 2026-05-08

**Authors:** Muhammad Tanveer Altaf, Aisha Umar, Mustansar Mubeen, Amjad Ali, Talha Shafique, Muhammad Asif Shabbir, Yasir Iftikhar, Muhammad Ahmad Zeshan, Manoj Kumar Solanki, Ahmed Mahmoud Ismail, Hossam S. El-Beltagi

**Affiliations:** 1 Department of Field Crops, Faculty of Agriculture, Recep Tayyip Erdoğan University, Rize/Pazar, Türkiye; 2 Institute of Botany, University of the Punjab, Lahore, Pakistan; 3 Department of Plant Pathology, College of Agriculture, University of Sargodha, Sargodha, Pakistan; 4 Department of Plant Protection, Faculty of Agricultural Sciences and Technology, Sivas University of Science and Technology, Sivas, Türkiye; 5 Department of Knowledge Research Support Service (KRSS), University of Management and Technology, Lahore, Pakistan; 6 Department of Life Sciences and Biological Sciences, IES University, Bhopal, India; 7 Pests and Plant Diseases Unit, College of Agricultural and Food Sciences, King Faisal University, Al-Ahsa, Saudi Arabia; 8 Agricultural Biotechnology Department, College of Agricultural and Food Sciences, King Faisal University, Al-Ahsa, Saudi Arabia

**Keywords:** bioremediation, fuel cell, heavy metals, mitigation, mycorrhizal fungi

## Abstract

Heavy metal contamination of soil and water is an escalating global environmental issue driven by industrialization and poor waste management. This problem is no longer confined to urban areas, as even small towns are grappling with severe heavy metal contamination, posing substantial threats to human health and aquatic ecosystems. To address these issues, fungal fuel cells have become one of the most promising and environmental friendly technologies. This technology is at the forefront of efforts to combat heavy metal contamination. This method utilizes the unique properties of fungi at the biocathode to treat contaminants and remove heavy metals from soil and water. In addition to reducing pollution, this technology has the capacity to generate electrical current which can serve as an alternative to conventional remediation methods. This review aims to provide a general overview of the fungal fuel cells as a method of bioremediation to remove toxic heavy metals and while simultaneously generating electricity. This review is based on a critical analysis of the recent peer-reviewed publications focusing on the development, operation, and application of fungal fuel cells in heavy metal remediation and bioelectricity production. By exploring the potential of fungal fuel cells, the review provides insight into a future where heavy metal pollution is effectively curtailed while contributing to sustainable energy production, thereby fostering a cleaner and healthier environment.

## Introduction

Certain heavy metals (HMs) in trace amounts are necessary for life, but as their concentration increases, they become poisonous to human health, animals, and aquatic life (Moncur et al., 2005). Exposure to environmental or natural concentrations of heavy metals is hazardous to various forms of life (Pavesi and Moreira, 2020). Heavy metal toxicity poses significant dangers to the environment, be it soil, air, or water, as these contaminants are released by activities such as chrome-plating, steel manufacturing, anti-corrosion agents, leather tanning industries, textiles, dyes, and pigments (Gu et al., 2015; Mala et al., 2015). Elevated metal concentrations can cause allergic reactions, carcinogenic effects, and enzyme dysfunction. Some heavy metal compounds are mutagenic, carcinogenic, and severely detrimental to ecosystems. Heavy metals can interact with red blood cells (RBCs), which may bind to the components of the cells because of the bioavailability of the metals (Chatterjee, 2015). Microorganisms have emerged as a promising tool for bioremediation, demonstrating their potential to restore wastewater, soil, and solid waste. This methodology is cost-effective and minimizes the need for invasive procedures (Mani et al., 2017). Microbial remediation can be conducted on-site and integrated with physical or chemical methodologies. It is recognized as a safe and efficient technological approach (de Alencar et al., 2017; Dixit et al., 2015). Combining microbiological methodologies with other techniques has become a central area of investigation in remediation research. Currently, there is growing interest in using fungal fuel cells in conjunction with other complementary treatment methods to enhance the elimination of heavy metals in polluted water and wastewater. Fungal fuel cells play a crucial role in the bioremediation process. Many fungal species can transform toxic forms of heavy metals into less toxic ones (Umar et al., 2023). The presence of metabolically active, redox-capable fungi is essential for the development and effective operation of fungal fuel cells for bioremediation. This review outlines the reduction of toxic metal quantities to less toxic forms through an economical and eco-friendly technique. The findings from this review emphasize the importance of Fungal Fuel Cells (FFCs) in converting metals from toxic to less toxic states while generating power. Single and double-chambered fuel cells effectively eliminate heavy metals (Mathuriya and Yakhmi, 2014). Dual-chambered FFCs or hybrid systems are more efficient than single-chamber systems waste treatment. FFCs represent a highly sustainable emerging technology for removing heavy metals while generating electricity. FFCs reactors efficiently treat heavy metals and various factors like microorganisms, electrode catalysts, structural design, metal concentration, cathode diameter, co-existing species, salinity, aeration, electron acceptor/donor, electrical connection type, electrolyte nature, resistance, temperature, pH, and carbon have noticeable effects on FFCs performance in metal transfer. The nature of fungi enhances degradation efficiency and electricity production. Microbial fuel cells (MFCs) have evolved to encompass various subfields, including power production (Munoz-Cupa et al., 2021), fuel manufacturing (Gebreslassie et al., 2021; Yang et al., 2021), odor mitigation, reservoir recovery (Zheng et al., 2015), energy storage (Qiu et al., 2021), sensors (Dai et al., 2021), and pollution mitigation (Xiao et al., 2021). Over the past 15 years, significant progress has been made in enhancing FFCs performance. This review focuses on the removal of heavy metals in power generation. Several excellent review articles have been published on different combinations. However, a review dedicated explicitly to fungal FFCs for a broad range of heavy metal pollutants and their power generation capacity is rare. FFCs represent a burgeoning and promising technological advancement in renewable energy generation and waste treatment. Despite the numerous advantages offered by MFCs, this technology still presents notable limitations that hinder its potential for widespread adoption and utilization in various application domains.

## Threats of heavy metals

The presence of heavy metals poses significant hazards to both the environment and human health. These toxic substances, including lead, mercury, cadmium, and arsenic, enter ecosystems through various pathways, such as industrial processes, mining activities, and improper disposal of waste. Once released, these elements accumulate in soil and water, entering and contaminating food chains and water sources. Exposure to heavy metals can result in severe health problems, including neurological disorders, developmental issues in children, and various types of cancer. Furthermore, heavy metal pollutants harm aquatic life, disrupt ecosystems, and may cause long-term environmental damage. Therefore, managing and mitigating the risks associated with heavy metals is crucial to protect the environment and human well-being.

## Chromium (VI)

Cr(VI) is a highly toxic environmental pollutant that has significant implications, thus requiring immediate remediation (Jobby et al., 2018). The pioneering investigation of Cr(VI) reduction within MFCs through electrochemical reactions marked a crucial breakthrough in environmental science. This pioneering study by Wang, ushering in a new era of research and innovation in the pursuit of mitigating Cr(VI) contamination. The potential of MFCs as a potent tool for addressing Cr(VI) pollution (Wang et al., 2018). This discovery has stimulated further studies to refine and optimize the use of MFCs in Cr (VI) remediation. Given that Cr(VI) poses severe threats to ecosystems and human health, the insights and findings from this research continue to inform and inspire further investigations, offering hope for more effective and sustainable solutions to combat this persistent environmental challenge.

## Iron (III)

*Fe(III)* has garnered considerable attention as a sustainable and cost-effective component in MFCs. Its pivotal role in enhancing MFC performance while being environmentally friendly has been widely acknowledged. He et al. (2017) conducted a study that focused on illuminating the role of *Fe(III)* as a mediator in MFCs and its influence on accelerating the reduction of *Cr(VI).* This research aimed to unravel the underlying mechanisms of *Fe(III)* mediation and its impact on catalyzing the reduction of *Cr(VI).* Fe(III) was observed to improve the reduction of chromium as well as the productivity of cathodic coulombic efficiency (CE). This paper demonstrates the significance of *Fe(III)* in microbial fuel cells and the possibility of its sustainable use in remediation of *Cr(VI)* by reduction, immobilization or recovery, rather than degradation.

## Silver

Silver, a valuable metal in nature, can be found in the waste products of industrial processes (Birloaga and Vegliò, 2018). Silver possesses numerous advantageous characteristics and attributes, including commendable electrical and thermal conductivity and remarkable malleability and ductility. When silver is recovered from the industrial wastewater, it is crucial for economic and environmental benefits due to its restricted availability and the diminishing availability of natural resources (Ho et al., 2018). The removal of silver (I) from wastewater is commonly achieved through various conventional methods, including adsorption, chemical precipitation, biosorption, and bioreduction. MFCs are cost-effective and have demonstrated silver removal efficiencies ranging from 99.91 to 98.26% when initial concentrations of silver ranged from 50 to 200 ppm. The MFC achieved a large power density of 4.25 W/m^2^ after 8 h (Choi and Cui, 2012). The process of silver recovery from wastewater in the presence of ammonia has been demonstrated (Wang et al., 2013). A sizeable electrical energy of 3.2 Joules was produced, resulting in 1.6 g of pure silver deposition on the cathode (Wang et al., 2013). Simultaneously, 1 g of COD (83%) was removed from the anode solution. Researchers have investigated the advancement of a profitable MFC system designed to retrieve silver metal from wastewater containing silver (Ag) ions (Hirooka and Ichihashi, 2013). The silver metal was recovered and accomplished with remarkable efficiencies ranging from 99.91 to 98.26%. Moreover, the silver output rate was 69.9 kg silver/kWh of energy output. The experimental setup involved the utilization of a part-feed cathode and a system of a continuously fed anode, wherein the initial silver concentration was set at 200 ppm.

## Copper (Cu)

The growing disparity between the global demand for copper and its supply has underscored the significance of extracting and recovering copper from diverse sources such as radioactive wastes and industrial effluents. This became imperative from an economic standpoint and environmental preservation (Ahmed et al., 2016). Moreover, copper is widely recognized for its significant toxicity and capacity to induce detrimental biochemical consequences in the human body, necessitating its removal. A maximum power density of 0.43 W/m^2^ was achieved under the anaerobic conditions in the chamber of the cathode, resulting in removal capabilities of approximately 99.88% (Miran et al., 2017).

## Cobalt (Co)

Cobalt, an indispensable cofactor for enzymes in all living organisms, possesses significant biological significance (Lwalaba et al., 2017). However, excessive concentrations of cobalt can pose substantial health risks to living organisms and ecosystems due to its toxic properties. An overabundance of cobalt exposure could lead to various diseases, including contact dermatitis, asthma, dermatitis, lung cancer, and pneumonia (Pimentel et al., 2014). According to Nancharaiah et al. (2015), Cobalt (III) serves as a highly effective terminal electron acceptor in the microbial fuel cells (MFCs). Huang et al. (2013), successfully described the leaching of cobalt from LiCoO2 in the MFCs by using acetate as a substrate, facilitated by an anode that had been acclimated. Liu et al. (2012), achieved significant enhancements in the leaching of cobalt (by 308%) and acid utilization efficacies (171%) in FFCs through the introduction of a *Cu(II)* concentration of 10 mg/L.

## Vanadium V(V)

Vanadium, in its pentavalent form [V(V)], is a highly versatile element applied in diverse domains. The global production of vanadium stands at approximately 38,000 metric tonnes per annum. An eminent utility of vanadium, as explicated by Weckhuysen and Keller (2003), is its role in enhancing the strength of steel. Its capacity to augment the resilience of steel against vibrations and shocks. Hence, vanadium is a much-coveted additive in steel manufacturing, contributing to the durability and potency of steel structures in various industries. Besides its industrial significance, vanadium is also acknowledged as a significant micronutrient. Research conducted by Mukherjee et al. (2004), has illuminated the association between vanadium and various human diseases, thus highlighting the multifarious nature of this element. The duality of vanadium’s roles as an industrial enhancer and a micronutrient with potential health implications underscores the need for comprehensive research and responsible management of its applications. Given that vanadium plays a pivotal role in diverse sectors, including metallurgy and health sciences, continuous studies and assessments are imperative to harness its benefits while addressing any potential risks or adverse effects associated with its use.

## Mechanism of HMs remediation

**Figure 1:**
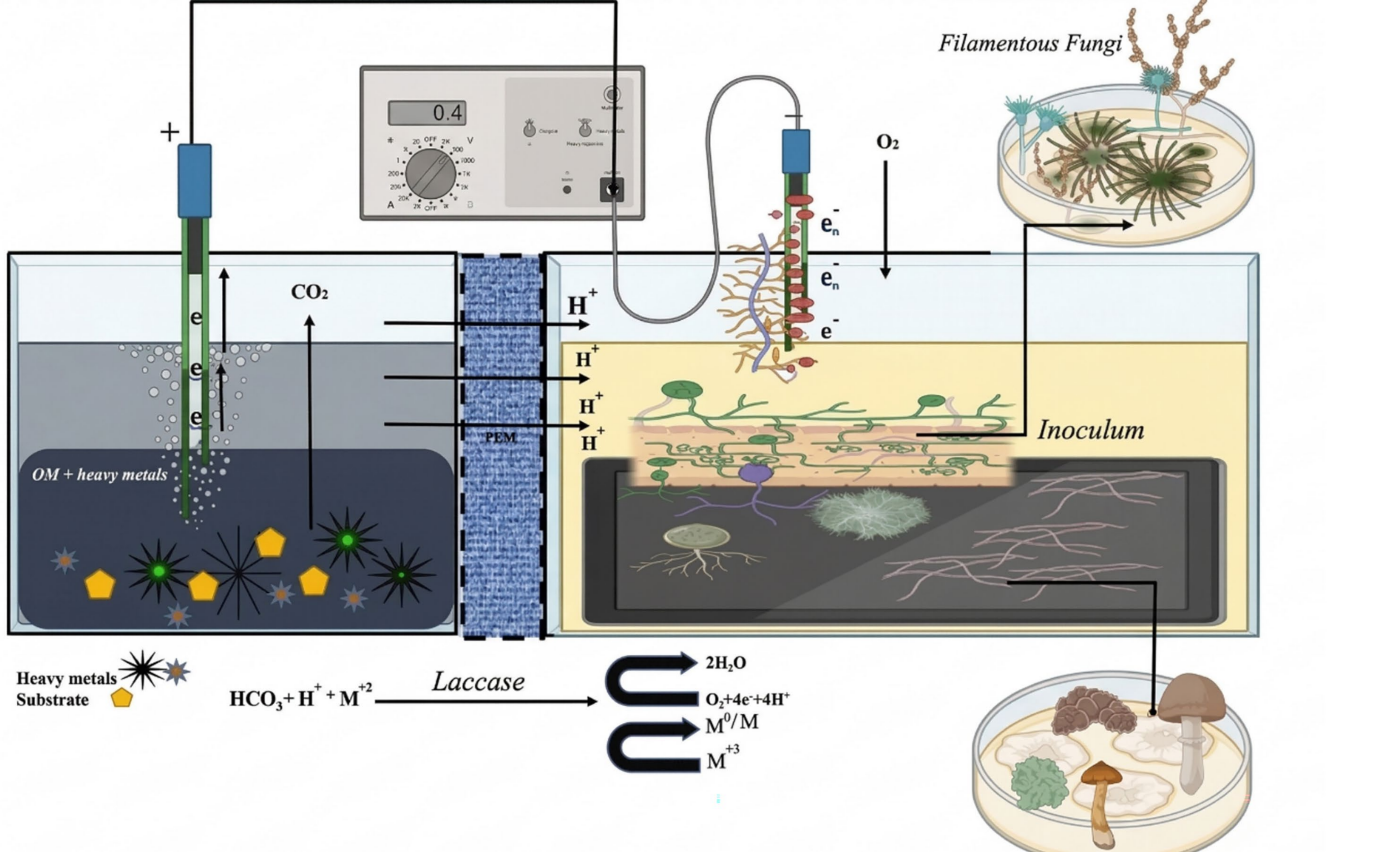
Presents a novel, environmentally conscious solution to heavy metal pollution through double-chambered fungal fuel cells. These fuel cells utilize filamentous and wood-rotting fungal catalysts to remediate pollutants while concurrently generating electrical power. This pioneering technology provides a dual advantage of pollution management and sustainable energy production.

Fungi have employed various mechanisms to circumvent the challenges and harmful effects linked to diverse heavy metals (de Alencar et al., 2017). Fungus employ various mechanisms for intracellular and extracellular sequestration, reduction, and enzymatic detoxification sensitive to the metal ions. After remediation of pollutants, the resulting products may be water and carbon dioxide or generate metabolic reactions that serve as primary substances for development (Figure 1). HMs cannot undergo direct degradation to transform into harmless compounds. Nevertheless, the mobility, chemical form, bioavailability, and toxicity of HMs could be altered by fungi via their growth metabolism and metabolic byproducts (Dixit et al., 2015). Typically, oxidation of organic matter occurring at the anode is associated with an oxygen reduction reaction at the cathode (Ucar et al., 2017). The primary role of the anodic chamber was to degrade the organic material, which served as both the carbon source and an electron donor (Gan et al., 2015). Heavy metal removal occurs in the absence of oxygen. Nevertheless, reducing any compound at the cathode with a redox potential equal to or greater than oxygen is possible. Consequently, various heavy metals are shown as effective electron acceptors in the cathode of FFCs (Modestra et al., 2017). This phenomenon could be attributed to the thermodynamic favorability of reducing heavy metal compounds. Consequently, the electron transfer from the anode to the cathode occurs continuously without external power input (Wang and RenZJ, 2014). The investigation for the elimination of heavy metals is still ongoing. Consequently, limited research has been conducted in this field. Tandukar et al. (2009), were among the pioneering researchers who embraced the utilization of biocathodes to address the issue of Cr(VI)-contaminated wastewater treatment and obtained a large power density, 55.5 mW/m^2^. In addition to the mechanisms described above, Wu et al. (2017), demonstrated that heavy metal removal at biocathodes can be facilitated through other mechanisms, such as bioreduction, bioaccumulation, biosorption, and biomineralization.

## Fungi as catalysts for power generation at the cathode

The arrangement of the cathode site and the terminal electron acceptor in the cathode chamber plays a crucial role in electricity generation in FFCs. Oxygen is a widely recognized primary electron acceptor due to its ready availability. Various species of fungi are being employed as catalysts at the cathode in fungal-based MFCs. White rot fungi, specifically *Ganoderma lucidum* and *Trametes versicolor*, are notable species used due to their superior electricity generation capabilities. Enhancement of the efficacy of the cathode relies on the fungal population in the cathode compartment, which generates enzymes that serve as catalysts for redox reactions. Laccase, an oxidoreductase enzyme containing copper, is widely believed to possess biological significance due to its oxidation reactions and enhance their role in biodegradation processes. Laccase is a multicopper oxidoreductase enzyme has been identified as the source of laccase secretion. According to the findings of Shleev et al. (2005), it was observed that the laccase could take electrons from the molecular oxygen, thereby facilitating the breakdown of organic compounds. The fungi that cause white rot produce and metabolize the laccase enzymes, which facilitate the nutrient cycling in soil by breaking down lignin, an organic component, within the ecosystem.

Fungal laccases consist of four copper molecules, exhibiting a notably enhanced ability to engage in redox reactions involving organic and aromatic compounds through an enzyme-mediated mechanism. Using white-rot fungi to produce laccase *in situ* can be considered a viable and economically efficient approach for promoting sustainable application. The efficacy of employing in situ fungi “white rot” for the laccase excretion was examined to enhance the FFC’s effectiveness. The degradation of lignin occurs as a result of laccase production. Wastewater sludge was employed as a substrate in the anode, while laccase was in the cathode compartment, under optimal environmental conditions. The findings, therefore, indicate the capacity of microbial enzyme systems to produce electricity.

## Different fungal species used in FFCs

**Figure 2:**
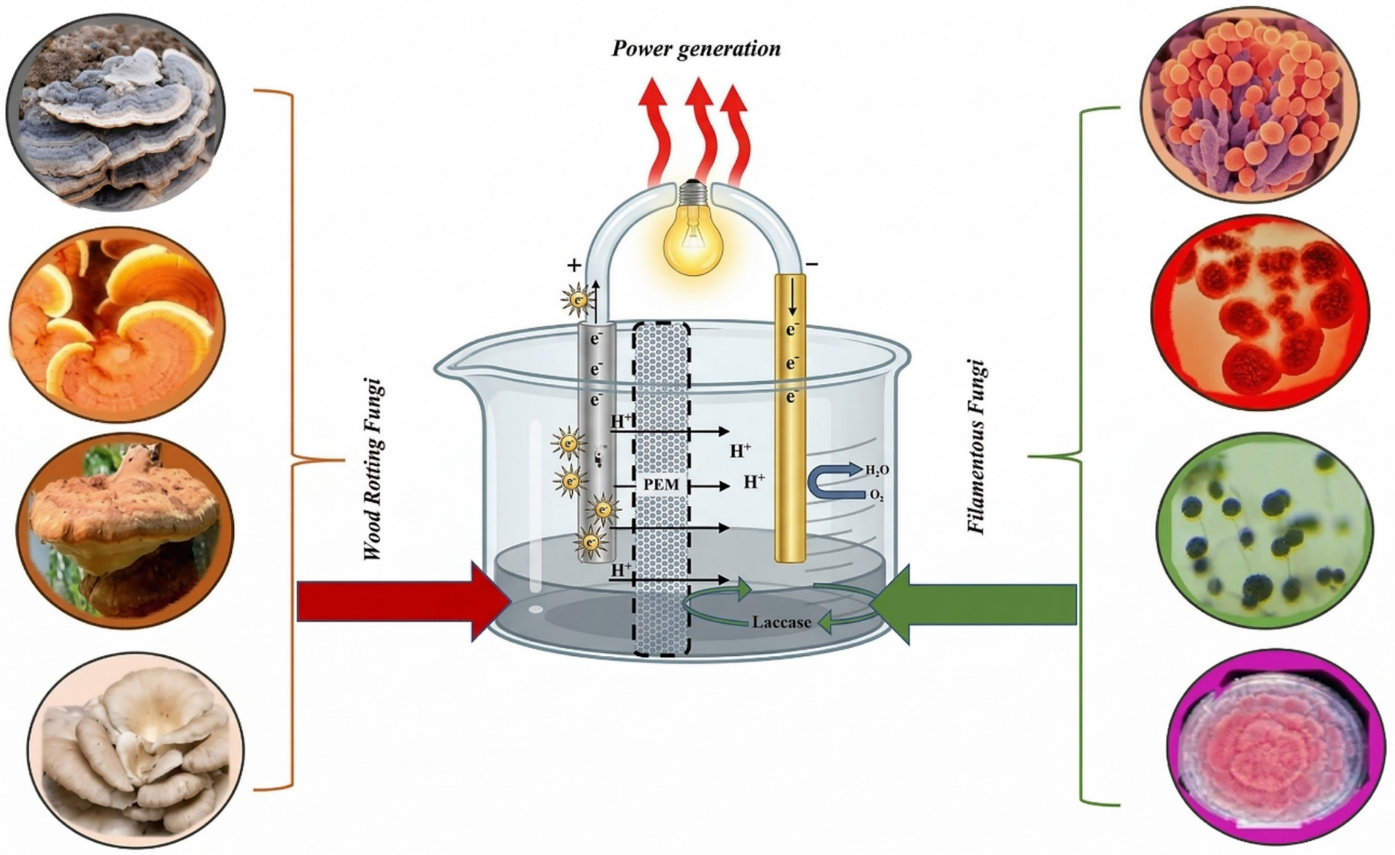
Highlights the critical role of diverse fungal species in addressing heavy metal contamination by secreting laccase enzymes, which are essential components in the degradation of metal contaminants. This process contributes to pollution control and generates electricity, thereby making it an eco-friendly and sustainable approach.

A broad spectrum of fungal species has garnered recognition for their potential catalytic role in fungal fuel cells (as depicted in Figure 2). These fungi exhibit unique metabolic capabilities that render them valuable contributors to the electrochemical processes within these fuel cells. By exploiting their enzymatic and redox reactions, these fungal species can convert organic matter, encompassing diverse pollutants and substrates, into electrical power. This catalytic capacity presents innovative pathways for sustainable energy production and bioremediation, thereby highlighting the versatile applications of fungal fuel cells in addressing energy and environmental challenges.

### Filamentous fungi

Various fungal species (*Aspergillus* sp., *Penicillium* sp., and *Rhizopus* sp.) isolated from soil samples were examined (Morant et al., 2014). These fungal species were utilized as a as cathodic biomaterials in the dual-chamber cathode compartment. This study employed cathode components to analyze energy outcomes in different fungal systems. In FFC, Pt-covered cathodes’ power generation reached 438.16 mWm^−3^, whereas cathodic carbon without Pt exhibited a power generation of 328.73 mWm-3. Nevertheless, the fungi, i.e., *Aspergillus* spp., achieved the highest energy output. As a biocatalyst, Laccase can facilitate the electron transfer towards the layer of the bacterial fuel cell from the cathodic electrode.

### Saccharomyces cerevisiae

*Saccharomyces cerevisiae* is a yeast species extensively utilized in contemporary biological approaches owing to its distinctive physiology and significant contributions to baking, food processing, and wine and beer production. Yeast is a highly effective biocatalyst in FFCs due to its capacity to metabolize various substrates. Yeast is suitable due to its simple cultivation, large-scale microbial production techniques, non-pathogenic nature towards non-target organisms, cost-effectiveness, rapid proliferation, and maintaining activity even in a desiccated state for extended durations. The significance of yeast cells in MFCs was highlighted by Christwardana and Kwon (2017), owing to their distinctive characteristics and sustainable nature. *S. cerevisiae* has been subject to comprehensive investigation and analysis as a biocatalyst within the context of biological fuel cells (Raghavulu et al., 2011). Yeast cell surface-displayed dehydrogenase has been utilized in various applications, such as pyranose dehydrogenase and cellobiose dehydrogenase (CDH) in the anodic compartment of FFCs (Gal et al., 2016). Different strains of yeast, including *Saccharomyces cerevisiae* (Gunawardena et al., 2008), *Pichia anomala* (Prasad et al., 2007), *Candida melibiosica* (Hubenova and Mitov, 2010), *Blastobotrys adeninivorans* (Haslett et al., 2011), and *P. polymorpha* has been recognized as potential catalysts in fuel cells. These are currently being employed as biocatalysts in anode chambers.

The metals influenced the density of the yeast cells’ adhesive property to the surface of the electrode, as indicated by the analysis of the scanning electron microscopy (SEM) results. Kasem et al. (2013) examined the transfer of electron process that took place through the species confined to the surface at the anode without a mediator. The impact of modifying the anode on the activity of *S. cerevisiae* based FFC has been examined. Power density increases with current density. The power density for Co was noted, i.e., 20.2 mW/m2, whereas, for Au, it was observed to be lower at 12.9 mW/m2. The cell of *S. cerevisiae* immobilized on the carbon nanotubes and is used as a biocatalyst in the membrane-free FFC. Hence, improvement in the electron transfer was observed (Christwardana and Kwon, 2017).

### Candida melibiosica

*Candida melibiosica* demonstrated its potential in phytase activity (Hubenova and Mitov, 2010). This characteristic has led to its utilization as an active catalyst in experimental settings of various biological fuel cells, with or without a mediator. *C. melibiosica* can produce bioelectricity of 60 mWm^−3^ without an extracellular mediator. This study demonstrated a correlation between bioelectricity generation, yeast growth factor, and substrate availability.

The performance of *C. melibiosica* has been the subject of extensive research to enhance the overall efficiency of FFCs. A significant increase in power density was observed when using MB at an amount of 0.8 mM, with values ranging from 20 to 640 mWm2. It was reported by Babanova in 2011 that outer mediators have the potential to enhance electron transfer kinetics and mitigate cell catabolism (Babanova et al., 2011).

*The capacity of Candida* sp. IR11, used to transfer electrons and produce electrical energy, was conducted using a pure culture of glucose-fed single-chambered FFC. Despite the yeast strain’s capability to decrease the concentration of ferric iron, it was considered that it would still maintain its capability of being electrogenic. It was examined that Candida sp. IR11 was placed in a distinct chamber of an FFCs. The substrate was sludge water effluent. The maximum power density of 20.6 ± 1.52 mWm-2 was recorded with 91.3 ± 5.29%. COD elimination.

### Pichia species

*Pichia anomala* is a fungal species belonging to the Saccharomycetaceae family. This species is renowned for its capacity to generate electrical energy without a mediator (Prasad et al., 2007). *P. anomala*, which is used as a catalyst for glucose oxidation because its cells are commonly incapacitated on the surface of the anode through a combination of physical adsorption and covalent bonding. *P. anomala* has enzyme capacities like ferricyanide reductase and lactate dehydrogenase, which are also present in the bacterial extracellular membrane and play a role in electron transfer. *P. polymorpha* and *P. anomala* are frequently employed as reliable power generation sources*. P. polymorpha* can be bombarded onto graphite electrodes for utilization in FFCs. The fungus exhibits thermotolerance and can endure a temperature range of 30–50 °C.

### Blastobotrys adeninivorans

A non-traditional yeast, *Blastobotrys adeninivorans* exhibits dimorphism, the ability to thrive within a restricted pH range, and heightened alkaline sensitivity under higher temperatures (48 °C). The biocatalytic activity of *B. adeninivorans* in a dual compartment FFCs, without the mediator, was investigated by Haslett et al. (2011). The peak energy density was 28 mWm^−2^. The transfer of electrons was accomplished through the utilization of an endogenous mediator; it was naturally synthesized within the solution, e.g., 2, 3, 5, 6-tetramethyl-1, 4-phenylenediamine redox mediator as an anode mediator or KMnO4 as the cathode reducing reagent mediated a noteworthy enhancement in the energy voltage (1.03 ± 0.06 Wm-2). The observed phenomenon can likely be attributed to the utilization of cyclic voltammetry of a supernatant by *B. adeninivorans*. The FFC comprised of *B. adeninivorans* exhibited superior outcomes compared to *S. cerevisiae*.

The catalytic activity of various fungal species (*P. polymorpha, S. cerevisiae, K. marxianus, Kluyveromyces lactis, P. pastoris, Schizosaccharomyces pombe, and C. glabrata*) in a mediator free dual chamber (Kaneshiro et al., 2014). The yeast Kluyveromyces marxianus exhibits the oxygenated ability to engage in respiro-fermentative metabolism. This metabolic process simultaneously produces energy from xylose and fructose, and it holds potential advantages in wood waste management.

### Wood rotting fungi

#### Trametes versicolor

*Trametes versicolor* is renowned for its proficient synthesis of ligninolytic and laccase enzymes. Wu et al. (2012) reported on the efficacy of employing the white-rot fungus, *Trametes versicolor* in enhancing the efficiency of an FFCs. Glucose was the exclusive carbon source for bioenergy generation in the cathode. To conduct a controlled experiment, one FFCs was enriched with laccase (catholyte), while the other FFCs was equipped with carbon fiber at the cathode; the abiotic cathode of the carbon exhibited a current range of 40 to 50 mWm-3. While using the white-rot fungi resulted in an elevated energy production (320 ± 30 mWm-3). The FFCs exhibited the greatest efficacy of conversion of 480 (± 30) mWm-3, when laccase catholyte was employed. The average energy output obtained from *C. versicolor* in the FFCs based on carbon fiber was two-thirds.

de Dios et al. (2013) examined the involvement of fungus in FFCs and their potential for energy production through sewage water and wastewater treatment. The biodegradation process accelerates by introducing highly capable fungal species, which play a significant role in the harmful toxicants that are remediation and energy generation. The presence of robust fuel cells obtained from microbes’ consortia has the potential to significantly impact both electron transfer processes and the degradation of biological substrates. In the present state of *Shewanella oneidensis*, the fungi *T. versicolor* exhibits the development of hyphae networks, facilitating the efficacy of electron transfer towards the anode. Forming the same genetic makeup of the microorganisms and fungi biofilms on the electrode requires approximately 30 days for FFCs activity. MFCs that are fortified with fungi exhibit a higher degree of utility.

#### Ganoderma lucidum

*Ganoderma lucidum* is a fungus species classified as an ornamental fungus of the family Ganodermataceae. This fungus is a prevalent white rot fungus that can decompose various hardwood materials. Lai et al. (2017), examined the utilization of this species in FFCs for degrading dyes. They focused on enhancing electricity generation by inoculating FFCs with laccase-producing fungi, specifically *Ganoderma lucidum*, onto the cathode surface.

#### Phanerochaete chrysosporium

Fungus *Phanerochaete chrysosporium* exhibits white-rot characteristics capable of degrading dyes through the enzymatic activity of laccase. Laccase catholyte in the FFCs is responsible for the degradation of azo dyes (Lai et al., 2017). Dyes were permitted to disperse from the anode compartment into the cathode chamber. Using PEM enhances fungi’s ability to access dyes via mycelium at the cathode. The findings from the experiments suggested that the biocathode based on fungus exhibits significantly superior performance compared to the conventional abiotic cathode, with a difference of almost 7 orders of magnitude (Wu et al., 2012).

#### Galactomyces reessii

*Galactomyces reessii* possess the capability of wood degradation through the production of laccase. The efficacy of dual chamber FFCs utilizing *G. reessii* and substantiated its significance as a biocathode based on the fungi (Chaijak et al., 2018). During the experiment, the production of laccase was observed. The utilization of coconut fiber was found to have a stimulating effect on the development of *G. reessii* and its laccase production. This resulted in attaining a maximum power generation voltage intensity of 253 mAm − 2 and efficiency of 59 mWm − 2 and within the FFCs. The study’s results indicated that using *G. reessii* as a biocathode material yielded notably intensity, greater peak strength, and energy values compared to using a Pt coated cathode in MFC.

Laccase enzymes obtained from *G. lucidum*, *T. versicolor,* and *Pleurotus ostreatus* have demonstrated notable success in energy generation by immobilizing fungal enzymes on a cathode surface (Mani et al., 2017). Laccase facilitates the replenishment of soil nutrients by catalyzing the breakdown of lignin-derived from plant material. Utilization of laccase as a biocathode resulted in a substantial reduction. The utilization of laccase in dual-chamber MFC results in a significantly higher power output (59 mW/m2) than in a single-chamber FFCs. ABTS [2,2′-Azino-bis (3-ethylbenzthiazoline-6-sulfonic acid)] is a proficient mediator for facilitating electron transfer from the electrode towards the laccase (Wu et al., 2012). The process of ABTS oxidation by white rot fungus is quantified through spectrophotometry at 420 nm. The power density and maximum voltage were 180 mV and 320 mW/m3. The initial voltage was low in the absence of ABTS. However, upon introducing specific quantities of ABTS, the voltage increased and enhanced the performance of fungus-based FFCs. For the optimal growth of fungal cells in a dual-chamber, it is necessary to have an airtight anodic compartment containing (100 mM) hexacyanoferrate at pH = 6.5, as well as a chamber of the cathode filled with a suitable growth medium that supports the secretion of laccase by a white-rot fungus.

## Fuel cells with and without mediator

In a mediator-less FFC, *S. cerevisiae* facilitates electron transfer to the surface of the anode via solution-phase species or surface-confined mechanisms (Sayed and Abdelkareem, 2017). In the absence of mediators, anode performance decreased from 0.4 to 0.1 volts over 24 and 21 h. Meanwhile, the open-circuit voltage (OCV) exhibits an enhancement of 0.25–0.65 V within a similar timeframe. Linear sweep voltammetry, a technique commonly employed to assess electrical activity at a working electrode, generates a peak power exceeding three mW/m^−2^. The power output may be limited by impeding the electron transfer rate from the microorganism to the anode surface (Christwardana and Kwon, 2017). Fungal strains are categorized according to electron transfer mechanisms via mediator-driven FFCs and mediator-less FFCs. Exogenous mediators, e.g., neutral red (NR), methylene blue (MB), bromophenol blue (BPB), bromothymol blue (BTB), cresol red (CR), bromocresol green (BcG), bromocresol purple eosin, methyl orange, methyl yellow, eriochrome black, murexide and methyl red are employed to enhance the electron transfer between the anodes and microorganisms.

## Electrode modification and performance of FFCs

Fungal fuel cells represent an innovative technology that can treat the various types of wastewater by harnessing the chemical energy present within them and converting it into electrical energy through active biocatalysts. The choice of electrode material significantly influences the performance and efficacy of FFCs. Different electrode materials, like carbon cloth, graphite, cobalt (Co), gold (Au), and carbon nanotube platinum (CNT/Pt)-coated CP carbon paper, have a significant impact on the performance of a dual-compartment MFC. The electrode’s performance was evaluated by measuring its half-cell potential and the power output of the fuel cell. The Co modification improved performance, whereas Au reduced it.

Fungal fuel cells, despite their environmentally friendly nature, require substantial investment and ongoing maintenance expenses, thereby compromising the economic viability of their implementation. The advancement of economically viable bio and abiotic electrodes has shown promise in reducing the costs of establishing FFCs. However, further investigation is needed to improve the design of these electrodes. Furthermore, these systems reduce energy consumption and facilitate the extraction of valuable constituents from wastes (including gold, heavy metals, and silver).

Fungal fuel cells have demonstrated remarkable efficiencies in the elimination and retrieval of heavy metals. This FFCs achieved an impressive *Cd(II)* removal efficiency (95%) by a maximum power density of 36.4 Mw/m^−2^ (Hasan et al., 2021; Shahat et al., 2021; Zhang et al., 2018). Likewise, significant progress has been made in developing functionalized adsorbents capable of effectively detecting metal contaminants like cesium, copper, and lead. These adsorbents have exceptional adsorption capacities (Hasan et al., 2021; Salman et al., 2021). MFCs have demonstrated removal rates of 98.3 and 89.6% for Cu2 + and *Pb(II)*, respectively (Rajendran et al., 2021; Wu et al., 2018).

## Synergistic approaches with FFCs

Synergistic approaches involving physiochemical techniques and microorganisms are predominantly employed in bioleaching and bio-stabilization. To mitigate these challenges, researchers have endeavored to optimize the efficacy and durability of bioremediation by establishing an appropriate ecological setting, implementing immobilization techniques, and providing a consistent nutrient supply. Lai et al. (2017), found that immobilization was efficient for stabilizing HMs during treatment. HM stabilization efficiencies (such as Cd, Cu, Pb, and Zn) were found to be more significant. Moreover, HMs have the potential for multiple reuses, thereby mitigating secondary pollution. The precise mechanism of stabilization remains uncertain. The primary mechanisms microbial cells interact with HMs include bioaccumulation, biosorption assimilation, bioleaching, biodegradation, biosynthesis, precipitation, and biotransformation.

## Factors influencing fungal fuel cells performance

The operational and environmental parameters that affect the performance and efficiency of fungal fuel cells (FFCs) are varied. These factors should be understood to maximize the removal of heavy metals as well as the production of electricity.

pH: pH is important in the control of fungal metabolism, enzyme activity and electron transfer. Most fungi grow best under slightly acidic to neutral environments that enhance enzyme activity, such as laccase production. The high pH levels may prevent the activity of microorganisms and decrease the remediation performance and electricity production (Wu et al., 2012; Ucar et al., 2017).

Temperature: The temperature has a strong influence on fungal growth and metabolism. The optimal temperature (generally 25–35 °C) boosts enzymatic activity and electron transfer rate, whereas changes may decrease system performance and reduce bioremediation efficiency (Cusick et al., 2012; Lu et al., 2017).

Substrate type: The type of substrate employed in the anodic chamber has a direct impact on the microbial activity and electricity production. Carbon sources and electron donors include organic materials, including wastewater sludge, which stimulate electron generation, but complex substances might take a longer time to break down (Gan et al., 2015; Flimban et al., 2019).

Electrode material: The composition of the electrode and surface properties are important in effective electron transfer. Carbon cloth, graphite, and carbon nanotubes are the materials that increase the conductivity and biofilm formation, thus promoting the overall FFC performance (Christwardana and Kwon, 2017; Ge et al., 2016).

Metal concentration: There is a direct correlation between fungal activity and system performance and the level of heavy metals. While moderate levels can be treated, high levels of it can cause microbial metabolism to slow down and interfere with the electron transfer (Huang et al., 2013; Rajendran et al., 2021).

Biosorption: Biosorption, bioaccumulation, and biotransformation are some of the mechanisms involved in fungal fuel cell that are at the center of the heavy metal removal mechanisms but can also be considered part of the system. In biosorption, the soluble heavy metals are bound to the surfaces of fungal cells by binding different metal to a variety of functional groups (e.g., carboxyl, amine, hydroxyl, and sulfonate) through electrostatic interactions, chelation, ion exchange, and complexation (Ahmed et al., 2016; de Alencar et al., 2017). Bioaccumulation enables the metals to be taken intracellularly and biotransformation enables them to be changed into a less toxic or less soluble form through redox reactions. These redox reactions are intimately connected to electron transfer in fungal fuel cells, in which electrons are liberated in metabolic activity or in reducing metal are transferred to the electrode, and play their part in the production of electricity. These processes, therefore, facilitate heavy metal recovery and bioelectricity generation.

Bioaccumulation: Bioaccumulation is a biological process where living organisms actively transport and store substances within their cells (Tabak et al., 2005). This process not only helps in the removal of metals in fungal fuel cells but also in the operation of the system. Metabolic and enzymatic redox reactions are generally linked to the intracellular uptake of metals, which may give out electrons in the process of transforming metals. These electrons are then passed on to the anode directly or through redox intermediates hence making a contribution to the production of electricity. Simultaneously, biosorption promotes the initial binding of metals to the cell surface whereas biotransformation transforms metals into less toxic or less soluble forms. These mechanisms are functionally combined in fungal fuel cells, which allow working on heavy metal remediation and bioelectricity generation at the same time.

## Bioassimilation

Bioassimilation involves the active transportation of siderophores by fungal cells. Microbes cannot acquire *Fe(III)* as a free ion. Therefore, microbes employ siderophores, low-molecular-weight chelating agents, to address this issue. These siderophores bind along with the iron and facilitate its transportation in the cell by an energy-dependent mechanism (John et al., 2001). In the meantime, certain metals, such as plutonium (Pu), can create siderophores with complexes. Numerous such complexes are acknowledged by proteins responsible for cellular uptake (Tabak et al., 2005).

## Bioprecipitation

It is a biogeochemical process that employs microbial metabolism to convert dissolved metal species into insoluble forms, such as carbonates, phosphates, hydroxides, and sulfides (Tsezos, 2009). Notably, microbial sulfides, especially those produced by sulfate-reducing bacteria (SRB), are highly efficient in immobilizing heavy metals (Tabak et al., 2005). This process holds significant potential for bioremediation strategies aimed at heavy metal-contaminated environments. Leveraging the innate precipitation capabilities of microorganisms, including SRB, enables the transformation of hazardous soluble metals into less mobile and less harmful forms, thereby mitigating their impact on ecosystems and human health. This process underscores the critical role of microbiology in developing sustainable environmental solutions.

## Biomobilization

Unlike organic contaminants, HMs are not susceptible to biodegradation. Alternatively, the speciation of HMs can be altered, for instance, using a biogeochemical mechanism, resulting in modifications to their mobility, toxicity, and bioavailability. The technique of biomobilization has been extensively employed for the remediation of contaminated sites with HM. The dissolved HMs are partitioned into distinct solid and liquid phases and subsequently subjected to further treatment.

## Bioleaching

The process by which metal minerals become soluble and the metals are free due to microbial activity. This methodology has been employed extensively to extract metals from mineral deposits. Indirect and direct mechanisms play a role in metal solubilization (Akcil et al., 2015; Gan et al., 2015). The direct leaching process involves the solubilization of metal sulfides, forming metal sulphates through enzymatic oxidation. On the other hand, in the indirect mechanism, microbes facilitate the oxidation of elemental sulfur or sulfur reduction compounds, leading to the production of sulfuric acid. Nevertheless, it is widely acknowledged that no established mechanism exists for directly oxidizing metal sulfide in biological systems. In contrast, the indirect mechanisms are responsible for the solubilization of metals from ores (Akcil et al., 2015; Vera et al., 2013). Bioleaching is a widely employed method for bio-mobilization, wherein the transformative properties of acid and biological oxidation generation convert undissolved metals into soluble ion forms. Furthermore, this method has shown promise in the remediation of contaminated soil, sludge, sediment and water (Gan et al., 2015). The microorganisms utilized in bioleaching primarily consist of chemoautotrophic fungi, e.g., *Aspergillus niger*.

Zeng et al. (2015) observed that the strain SY1 of *Aspergillus niger* demonstrated the ability to effectively eliminate Cu, Cd, Pb, and Zn from dredged sediments contaminated with various metals. Notably, the strain exhibited a removal rate exceeding 90%, with Cd being the metal most effectively removed. The research revealed that introducing H_2_O_2_ instigated a reaction resembling the Fenton process, eliminating additional metal contaminants. The removal of Cu, Pb, Cd, and Zn increased from 60 to 70%, 60 to 70%, 90 to 99.5%, 20 to 39%, and the integrated procedure effectively enhanced the dewatering capability of polluted dredged sediments. Finally, an excessive supply of carbon sources can lead to secondary pollution resulting from organic matter (Chai et al., 2009). In contrast to alternative methodologies, bioleaching reduced expenses, diminished energy demands, heightened environmental security, and enhanced operational adaptability (Zheng et al., 2015).

## Biodegradation

This involves oxidation of organic contaminants. Complex organic compounds degradation could have a substantial effect on the mobility of the heavy metals, their toxicity, and their bioavailability in the subsurface environments (Tabak et al., 2005). Acid tolerant microorganisms like *Rhodotorula* spp. and *Aspergillus niger* break down dissolved organic materials and the electrons are released in the metabolism. These electrons are passed on to the anode in fungal fuel cells by the extracellular electron transfer pathways, which adds to the production of electricity. At the same time, biosorption, bioaccumulation, and biotransformation mechanisms are used to bind, uptake, and redox transform heavy metals, making it possible to eliminate them and allow the flow of electrons within the system (Gan et al., 2015).

## Biotransformation

The redox reactions thereby altering their toxicity, mobility, and bioavailability, the toxicity, mobility, and bioavailability of the heavy metals. Metal reducing fungi can be directly reduced to insoluble and less mobile, *Tc(IV), Cr(III)*, and *U(IV)*, respectively, by soluble and mobile *Tc(VII), Cr(VI)*, and *U(VI)*. The diminished products can also develop increased metal-reduction indirectly (Tsezos, 2009). In fungal fuel cells, biosorption, bioaccumulation, and biotransformation mechanisms are important in the linkage of the removal of heavy metal and production of electricity. Biosorption allows the fungi to adsorb the metal ions onto their cell walls whereby the transfer of electrons to the anode may occur. Further bioaccumulation aids in intracellular uptake of metals with bioaccumulation, and redox reactions, biotransformation changes chemical state of metals, reducing toxicity and mobility. The combination of these microbial processes and extracellular electron transfer is what ensures that the heavy metals are removed and that electrical current is generated concomitantly, making FFCs an effective technology regarding sustainable bioremediation and energy recovery.

## Myco-phytoremediation of HMs

Fungi that can thrive in environments contaminated with harmful substances have evolved various mechanisms to alter the chemical composition of HMs. These adaptations ultimately affect the bioavailability of HMs (Sarwar et al., 2017). Using microorganisms to remediate heavy metals primarily involves the application of symbiotic and saprophytic mycorrhizal fungi (Philippot et al., 2013; Sarwar et al., 2017). Mycorrhizal fungi are a significant constituent of the soil microbial community in the root zone, establishing symbiotic associations with a wide range of higher plants in various manifestations (Sarwar et al., 2017). The symbiotic relationship between plant roots and fungi can have advantageous effects on plants, such as the facilitation of nutrient accessibility through the establishment of a comprehensive network of hyphae. According to (Sarwar et al., 2017), Fungi can alter the composition of root material, the pH of the soil, and the bioavailability of heavy metals in the soil. Arbuscular mycorrhizal fungi (AMF) are widely prevalent mycorrhizal fungi. They play a vital role in facilitating the uptake and accumulation of HMs in plants while also enhancing the plants’ ability to withstand the adverse effects of HMs. Such processes are additionally impacted by the specific fungi and plant species, as well as the characteristics of the soil (Merlos et al., 2016; Wu et al., 2016). There are two primary perspectives regarding fungi’s impact. Some studies have indicated that fungi can enhance the process of HM phytoextraction. Other studies have demonstrated that fungi can mitigate HM phytoextraction, promoting plant tolerance to HMs.

The introduction of arbuscular mycorrhiza *Glomus mosseae* to *Trifolium pratense*, L. enhanced plant yields and increased Zn phytoextraction (Chen et al., 2003). Moreover, *Glomus mosseae* can enhance exchangeable and carbonate-bound HMs in dredged sediment (Peng et al., 2018). This increased phytoextraction of HMs (such as Cr, Pb, Cd, and Zn) by maize, *Medicogo sativa* L., and *Lolium multiflorum* Lam. Moreover, Wu et al. (2016), observed that the extraradical mycelium of upon inoculation with *Rhizophagus irregularis*, exhibited the ability to absorb and transport Cr towards the fungal roots of *Taraxacum platypecidum* Diels. Nevertheless, the migration of Cr from the roots to the shoots was found to be restricted, resulting in the sequestration of Cr in the roots and alleviating the phytotoxic effects of Cr on the plant.

Merlos et al. (2016) observed two maize cultivars: the and the Cu-tolerant cv. *Oropesa* and Cu-sensitive cv. *Orense* exhibited elevated copper amounts after inoculation with *Rhizophagus irregularis*. The potential enhancement of Cu tolerance in the mycorrhizal plant cultivar Orense can be attributed to an augmented stimulation of shoot phytochelatin biosynthesis due to symbiotic interactions. Peng et al. (2018) reported that the growth of *Glycyrrhiza uralensis* Fisch can be enhanced by *Rhizophagus intraradices*. This enhancement was evident in various aspects, such as phosphorus, plant biomass, and chlorophyll levels. The application of biogas residue was found to significantly impact reducing the concentrations of Pb and Cu in *Glycyrrhiza uralensis* Fisch, with the involvement of *Rhizophagus intraradices.* Table 1 indicates the literature data collected on behalf of electricity production via fungal fuel cells during heavy metals mitigation.

**Table 1:** Removal of heavy metals and electricity generation in fungal fuel cells by a fungal fuel cell.

Heavy metals	Removal efficiency (%)	Power generation (mW/m^2^)	FCs type	References
Cr(VI)	99	150	Single	Fan et al. (2020), Wang et al. (2008), and Zhang et al. (2021)
Zn(II)	25	3.6	Three chamber	Abourached et al. (2014) and Ganiyu et al. (2020)
Cd(II)	18–31%	Not reported	Two chamber	Ganiyu et al. (2020) and Zhang et al. (2015)
Pb(II)	44.1	392	Two chamber	Zhang et al. (2015)
Cu(II)	99%	399	Single/double	Bhat et al. (2020) and Tao et al. (2011)
V(V)	87.9	578.3	Double chamber	
Hg	99.54	433.1	Double	Wang et al. (2011)
Se	99	392	Single	Catal et al. (2009)
Co	62.5	298	Double	Huang et al. (2013)
Ag	99	4.25	Tubular	Nancharaiah et al. (2015)
Au(III)	99.8	6.58	Tubular	Nancharaiah et al. (2015)
Toxic TI	67	457.8	Single	Wang et al. (2018)
Pt	90	844	Double	Modin et al. (2012)
Ni	Not reported	Not reported	Double	Liu et al. (2012)

Under different conditions of system design and operation, fungal fuel cells (FFCs) differ in their performance in terms of power density and heavy metal removal efficiency considerably. According to the reported studies, the removal efficiencies are typically high with some metals, including *Cr(VI), Cu(II),* Ag, and Hg, which are usually above 90 percent, and this proves the high potential of FFCs on effective bioremediation (Wang et al., 2008; Tao et al., 2011; Nancharaiah et al., 2015). Conversely, middle to low removal efficiencies have been reported with other metals such as *Pb(II)*, Co and *Zn(II)*, which indicate inconsistencies between the different types of metals and system setups (Zhang et al., 2015; Huang et al., 2013; Ganiyu et al., 2020).

The power density outputs also have a wide range with low values (e.g., 3.6 mW/m^−2^ with Zn) and relatively high outputs of over 500 mW/m ^−2^ when considering systems that are treating metals like vanadium and platinum (Abourached et al., 2014; Modin et al., 2012). Such differences depend on such factors as electrode material, design of the reactor (single or double chamber), and microbial activity. In general, though FFCs have excellent removal efficiency, power production is intermittent and can be rather low, which means that additional optimization is necessary to balance the performance of these systems in terms of remediation and energy generation.

## Current developments in biotechnology

**Figure 3:**
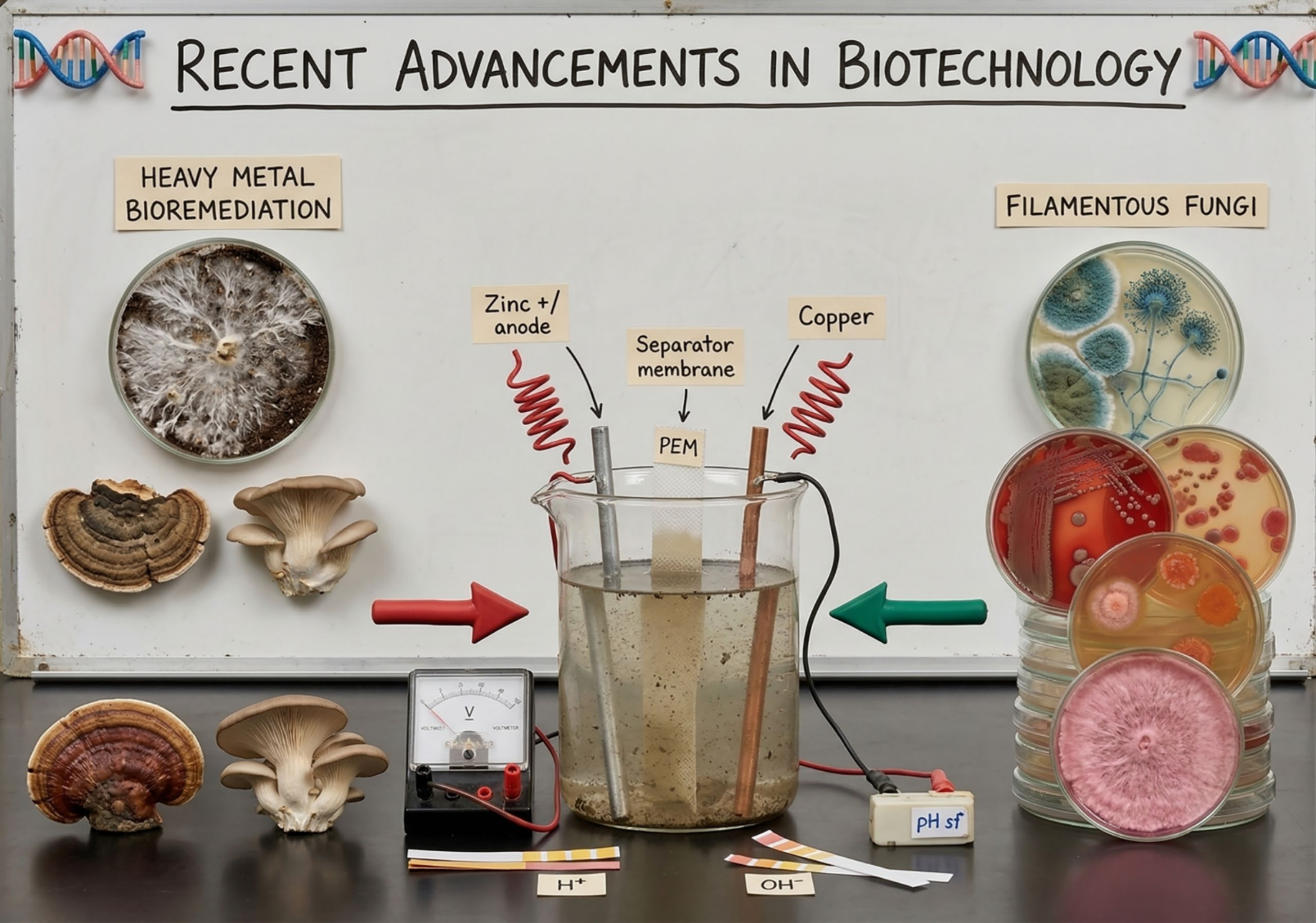
The latest uses of fungal fuel cells.

There is a breakthrough in biotechnological advancements, according to which fungal fuel cells (FFC) are used for electricity generation by catalyzing electrochemical reactions (Tiwari et al., 2021). FFCs use organic matter and convert it into electricity through a rigorous metabolic process. Electricity generation by using FFC has gained the vital attention of environmental scientists, as it may play a significant role in heavy metals amelioration (Raven et al., 2019). The flow chart below determines how FFCs may participate in electricity generation (Figure 3).

## Bioremediation of heavy metals by using fungi

Fungi are regarded as highly efficient decomposers of diverse organic compounds, participating in the processes of decay and destroying wood’s lignin and cellulose that are frequently discarded in the agricultural industry (Umar et al., 2023; Thatoi et al., 2014). Fungi excrete various enzymes within and outside the cells used in the metabolism of many compounds (Deshmukh et al., 2016). Almost all fungi’s biodegradation abilities are associated with oxidative enzymatic processes, which are mediated by various oxidases and peroxidases (El-Gendi et al., 2021).

## Fungi as a source of electricity generation

By oxidizing the inorganic chemicals in biomass, fungi act as catalysts in the fungal fuel cell, a device that produces energy (Lovley, 2006). Through the biodegradation of raw materials, fungal cells produce electricity directly for use in fungal fuel cells (FFCs) (Rabaey and Verstraete, 2005). Compared to conventional approaches, fungal cells have a nine-fold higher capacity to create energy stored in sewage sludge (Flimban et al., 2019).

## Wastewater treatment through Fungi

The microbial community of wastewater contains many fungi, which are highly valuable and usable due to their diverse functions (Hyde et al., 2019). Fungi may readily adapt to harsh conditions and quickly changing surroundings, such as various industrial and municipal wastewater types, heavily hydrocarbon-polluted sites, acid substrates, or low oxygen levels (Behnamia et al., 2018). Although the fungus population in wastewater settings varies greatly, a core group of similar genera has been documented (Zhang et al., 2018). The most prevalent species are *Penicillium, Candida,* and *Geotrichum*, followed by a more diverse group that includes *Rhodotorula, Trichoderma, and Trichosporon*. Season and temperature impact the variance of fungal taxa in treated plants as well; fungal diversity appears to change with the summer and winter seasons. In the warmer months, several taxa are more prevalent, including *Penicillium, Trichoderma, Acremonium, and Aspergillus* (Wei et al. 2018).

## Limitations of fungal fuel cells

Although fungal fuel cells (FFCs) have great potential, there are a number of limitations that inhibit their large-scale use and practical application. The major problem is low power output that is not enough to generate energy at commercial scale. Despite the reported improvements, the total energy output of FFCs remains rather low in comparison to traditional energy technologies (Cusick et al., 2012; Flimban et al., 2019). The other important constraint is connected with scale-up issues. The majority of studies are carried out at the laboratory level, and the challenges of translating these systems to the real world are technical and economic in nature, such as the complexity of reactor design and operational stability (Lu et al., 2017; Wang et al., 2020). The expensive price of electrode materials and membranes is also a significant impediment. Cathodes and proton exchange membranes represent significant cost factors in the entire system, which restricts economic viability (Ge and He, 2016). Also, fungal stability and longevity during the long run of operation is of concern. Fungal activity could be influenced by changes in environmental factors, availability of substrates, and even by toxicity caused by high levels of metals, decreasing the efficiency of the system over time (Christwardana and Kwon, 2017; Huang et al., 2013). To solve these limitations and achieve the development of FFC technology to practical and large-scale use, it is necessary to address them with better reactor design, cost-effective materials, and improved biological stability.

## Conclusion

Fungal fuel cells (FFCs) are one of the future perspectives of both combating heavy metal pollution and generating renewable energy, which is environmentally friendly. FFCs facilitate the conversion, confinement, and retrieval of toxic heavy metals with the conversion of bioelectricity by merging the enzymatic and metabolic properties peculiar to fungi with electrochemical systems. This two-fold capability makes the difference between FFCs and the traditional remediation technologies which can be energy-intensive and invasive to the environment. The review notes that fungal species especially laccase-producing and metal-tolerant strains are important when it comes to improving electron transfer and catalytic efficiency in fuel cells. The efficient removal of heavy metals is achieved through mechanisms like biosorption, bioaccumulation and biotransformation, whereas the ongoing electricity production is enabled by extracellular electron transfer pathways. Moreover, the improvements in the electrode material, reactor design, and mediator systems have enhanced the performance and applicability of FFCs by a great deal. Along with these positive aspects, there are still issues of low power density, high material costs, and limited large-scale implementation. Future studies should aim to fungi-electrode contacts more efficient, finding economical and scalable reactor designs and investigating hybrid reactor systems incorporating FFCs with other treatment methods. All in all, FFCs provide an attractive, eco-friendly product, which corresponds to the world needs of sustainable environmental mitigation and clean energy generation. As further technological improvements are made, fungal fuel cells can become a viable and effective tool in reducing heavy metal pollution and become a component of decentralized energy systems.

## Challenges and future pathway

### Efficiency improvement of fungi in energy generation

Future energy systems may use the fungal fuel cell approach instead of waste products to generate power from wastewater, which typically has organic contents in the much smaller range of 260–450 mWm^−2^ (Cusick et al., 2012). In the future, the ultimate goal is to design a cell that can produce the most energy at the lowest feasible energy input expenditure.

### Scaling up and real-world uses

The scale-up experiments have shown several difficulties. The cost-effectiveness of these MFCs would be the most critical factor. The scale-up system’s capital expenses vary from USD 735/m^−3^ to USD 36,000/m^−3^ (Wang et al., 2020). The leading causes of an MFC system’s high cost are the electrodes, particularly the cathode, and the membrane (Ge et al., 2016). Thus, developing affordable reactor components and configuration would be the advised course of action for scale-up. Membrane-free MFCs or MFCs with inexpensive membranes, such as dynamic membranes, glass fiber separators, and nano-filtration membranes, have gradually replaced membraned MFCs as the main subject of contemporary research. In addition to the initial investment, ongoing expenses like mixing and pumping wastewater have a negative impact on energy consumption (Lu et al., 2017).

### Low environmental impact

Unlike traditional restoration methods, which often involve harsh chemicals, fungal fuel cells operate in a manner that is more sustainable and kinder to the environment. It is a cleaner and greener method because it depends on natural biological processes. After all, the ecological impact of cleanup activities is reduced.

### Versatility and adaptability

The ability of fungi to adapt to various environmental conditions is well known. Fungal fuel cells are highly adaptable and can be utilized in multiple settings. Whether used in agricultural areas affected by heavy metal runoff or industrial wastewater treatment, fungus fuel cells can be tailored to satisfy specific pollution issues.

### Possibility for large-scale deployment

Because of its scalability, fungus fuel cells are a technology that can be implemented widely. In an era of an increasing need for sustainable and decentralized energy solutions, fungal fuel cells offer a workable alternative for environmental cleanup and electricity generation. They also contribute to developing a more resilient and clean energy infrastructure.
